# Fulminant Amyloid-β–Related Angiitis after Tenecteplase: A Biopsy-Confirmed Cause of Post-Thrombolysis Neurologic Deterioration

**DOI:** 10.1177/19418744261488454

**Published:** 2026-09-18

**Authors:** Christina Schweitzer, Kalkini Durai, Shreeja Kadakia, Grant Zeigler, Khoa Pham

**Affiliations:** 1 Department of Neurology, 12310 Penn State College of Medicine, Hershey, PA, USA; 2 Department of Pathology and Laboratory Medicine, 12310 Penn State College of Medicine, Hershey, PA, USA

**Keywords:** amyloid-β–related angiitis, cerebral amyloid angiopathy, thrombolysis, tenecteplase, intracerebral hemorrhage

## Abstract

**Background:**

Amyloid-β–related angiitis (ABRA) is a rare steroid-responsive inflammatory vasculopathy that can mimic or complicate acute cerebrovascular disease. Its interaction with thrombolytic therapy is poorly understood. We report a biopsy-confirmed case of fulminant ABRA temporally associated with intravenous tenecteplase.

**Case Description:**

An 86-year-old man presented with acute painless monocular vision loss and received tenecteplase for presumed central retinal artery occlusion. Initial imaging showed no hemorrhage. Within 24 hours, he developed progressive encephalopathy with a large left frontal lobar hemorrhage, additional contralateral lobar hemorrhages, and diffuse subarachnoid hemorrhage requiring surgical evacuation. Pathology demonstrated Congo red–positive amyloid deposition with transmural lymphocytic and macrophage-predominant inflammation and fibrinoid necrosis, confirming ABRA. MRI showed progressive vasogenic edema, new punctate infarcts, increasing cortical microbleeds, and convexity subarachnoid hemorrhage. Infectious evaluation was negative. High-dose corticosteroids led to gradual neurologic improvement.

**Conclusions:**

Thrombolysis may unmask or exacerbate inflammatory cerebral amyloid angiopathy, causing multifocal hemorrhage, ischemia, and rapid decline. In older patients with unexpected post-thrombolysis deterioration and evolving lobar hemorrhage, edema, and microbleeds, ABRA should be considered, as early immunosuppression may improve outcomes.

## Introduction

Amyloid-β–related angiitis (ABRA) is an inflammatory vasculopathy within the spectrum of cerebral amyloid angiopathy–related inflammation, characterized by amyloid deposition in cortical and leptomeningeal vessels accompanied by granulomatous, angiodestructive inflammation.^1^ Although classically presenting with subacute cognitive decline, seizures, or focal neurologic deficits, ABRA may mimic or complicate acute cerebrovascular disease. Diagnosis often requires brain biopsy to distinguish it from primary CNS vasculitis, and, unlike noninflammatory cerebral amyloid angiopathy, ABRA is frequently responsive to high-dose corticosteroids.^2^

The interaction between ABRA and acute thrombolytic therapy is poorly understood. We describe a biopsy-confirmed case of fulminant ABRA temporally associated with intravenous tenecteplase administration for suspected acute ischemic stroke, highlighting an underrecognized cause of post-thrombolysis neurologic deterioration.

## Case Presentation

An 86-year-old man with hypertension, hyperlipidemia, polymyalgia rheumatica, prediabetes, and a prior small ischemic infarct presented with acute painless left monocular vision loss. His NIH Stroke Scale score was 0. Blood pressure was 168/72 mmHg and serum glucose was 102 mg/dL. Noncontrast CT and CT angiography showed no acute infarct or hemorrhage but revealed 60%–69% stenosis of the left internal carotid artery. Given concern for central retinal artery occlusion, intravenous tenecteplase was administered. His premorbid modified Rankin Scale score was 1.

The patient’s prior small ischemic infarct was an incidentally identified late subacute punctate infarct in the left peritrigonal white matter on MRI from 7 months earlier. That MRI included susceptibility-weighted imaging and demonstrated several punctate foci of SWI signal loss, interpreted as chronic microhemorrhage vs small benign cavernoma. Following that evaluation, aspirin 81 mg daily and rosuvastatin 20 mg daily were initiated, and aspirin remained part of his home medication regimen at the time of presentation.

The patient’s presenting symptoms included last known well at 08:30, he subsequently developed sudden painless diffuse “fogging” of the left eye around 09:00. He denied trauma, headache, jaw claudication, flashes, floaters, curtain-like vision loss, diplopia, weakness, sensory loss, dysarthria, aphasia, dysphagia, or gait difficulty. Neurologic examination was otherwise nonfocal, with fluent speech, intact cranial nerves aside from left monocular visual impairment, full strength, intact sensation, and no appendicular ataxia. NIHSS was documented as 1 for left monocular visual impairment.

Given the sudden onset of potentially disabling monocular visual symptoms in the setting of ipsilateral carotid stenosis, central retinal artery occlusion was suspected. The patient confirmed that the visual deficit would be functionally disabling, including limiting his ability to drive. Risks and benefits of intravenous thrombolysis were discussed, including the risk of significant hemorrhage and angioedema, and the patient consented to treatment. Intravenous tenecteplase was administered at 10:39 after discussion with the vascular neurology attending, and the patient reported improvement in left eye vision within 30 minutes.

Subsequent ophthalmologic evaluation demonstrated left vitreous hemorrhage, with diffuse pigmented cells in the vitreous and no retinal detachment, giant retinal tear, or intraocular mass on ocular ultrasound. The etiology of the initial visual symptoms therefore remained uncertain, with primary vitreous hemorrhage vs suspected CRAO with subsequent hemorrhagic complication considered.

**Figure 1.**
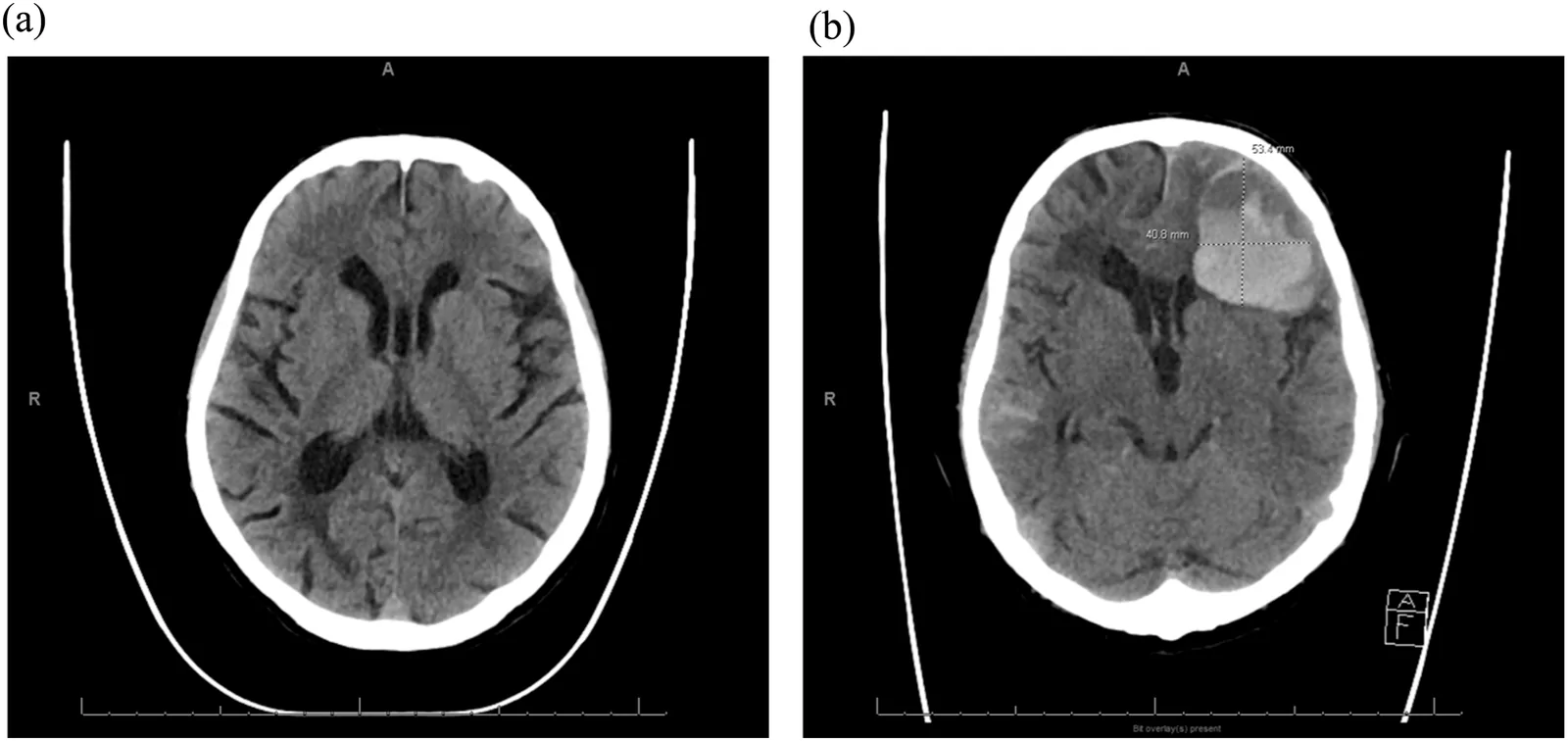
Head CT before and after tenecteplase administration. (A) Pre-thrombolysis noncontrast CT head obtained on 11/21/2025 10:21 demonstrated no acute intracranial hemorrhage, territorial infarct, extra-axial collection, or mass effect. (B) Post-thrombolysis noncontrast CT head obtained on 11/22/2025 demonstrated a large acute left frontal intraparenchymal hematoma, an additional right posterior parietal intraparenchymal hematoma, bilateral subarachnoid hemorrhage, intraventricular blood products, and associated rightward midline shift with subfalcine herniation

Within 24 hours, he developed progressive encephalopathy. Repeat CT demonstrated a large left frontal lobar intraparenchymal hemorrhage (∼39 mL), 2 smaller right parietal hemorrhages, and diffuse subarachnoid hemorrhage (Figure 1). He underwent surgical hematoma evacuation. Postoperatively, mental status remained depressed without electrographic seizures on serial EEG.

**Figure 2.**
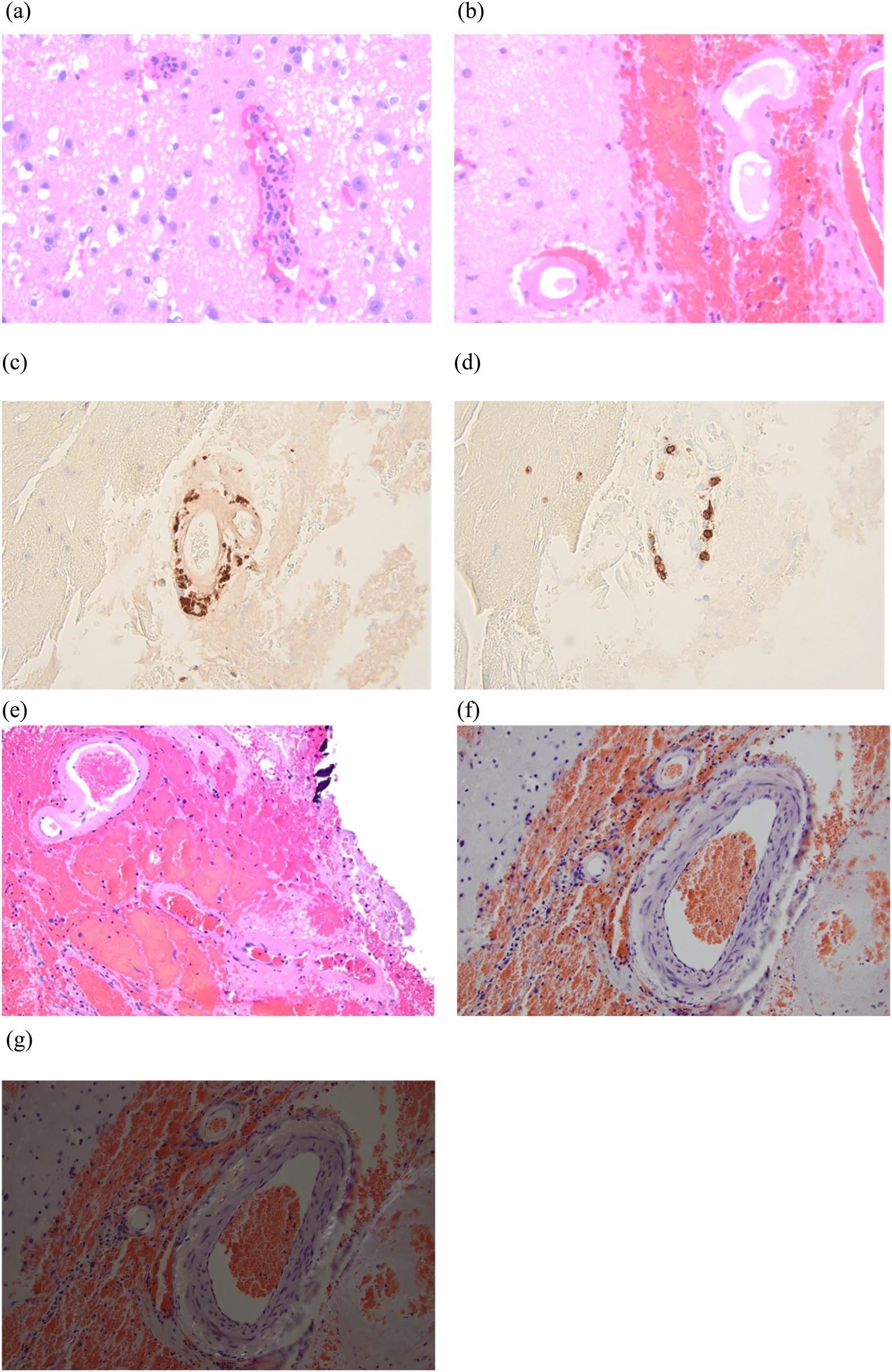
Histopathology from biopsy. (A) H&E 40×, showing fibrinoid necrosis and transmural inflammatory infiltration of vessels by lymphocytes and macrophages. (B) H&E 40×, demonstrating eosinophilic amyloid deposition within leptomeningeal and parenchymal vessel walls. (C) CD68 immunohistochemistry highlighting macrophage infiltration of the vessel wall. (D) CD3 immunohistochemistry demonstrating T-cell infiltration. (E) H&E 20×, showing additional areas of fibrinoid necrosis. (F) Congo red staining, 20×, positive for amyloid deposition in vessel walls. (G) Congo red under polarized light demonstrating apple-green birefringence

**Figure 3.**
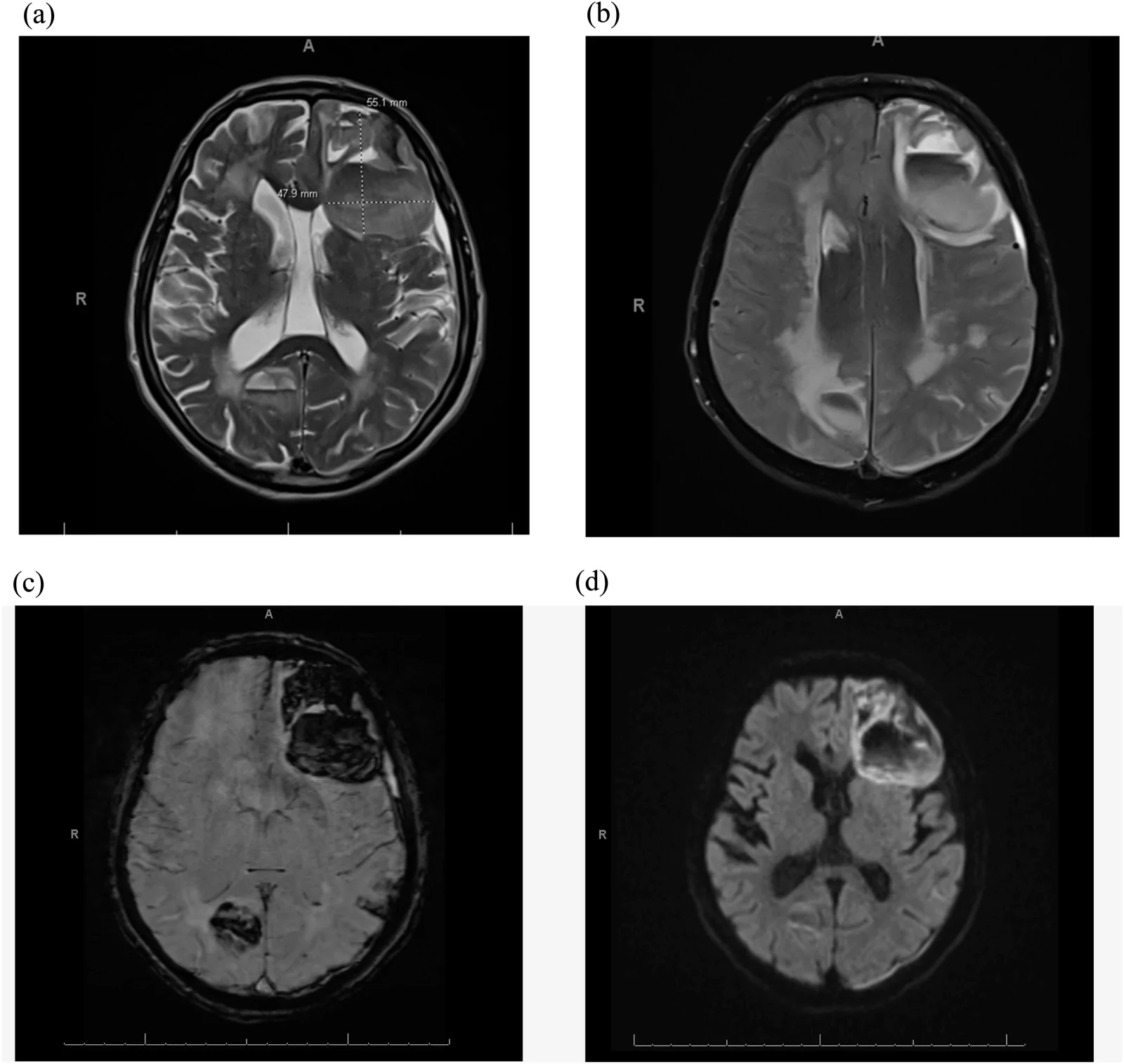
MRI findings on 11/22/2025 after tenecteplase administration. (A) Axial T2-weighted MRI demonstrated a large left frontal intraparenchymal hematoma with surrounding vasogenic edema and mass effect. (B) Axial T2/FLAIR MRI demonstrated the left frontal hematoma with adjacent vasogenic edema, additional white matter hyperintensity, and associated subarachnoid and intraventricular blood products. (C) Axial susceptibility-weighted MRI demonstrated susceptibility effects corresponding to multifocal intraparenchymal, subarachnoid, and intraventricular blood products. (D) Diffusion-weighted MRI demonstrated small cortical/juxtacortical foci of restricted diffusion in the high left frontal lobe, compatible with small acute to early subacute infarctions

**Figure 4.**
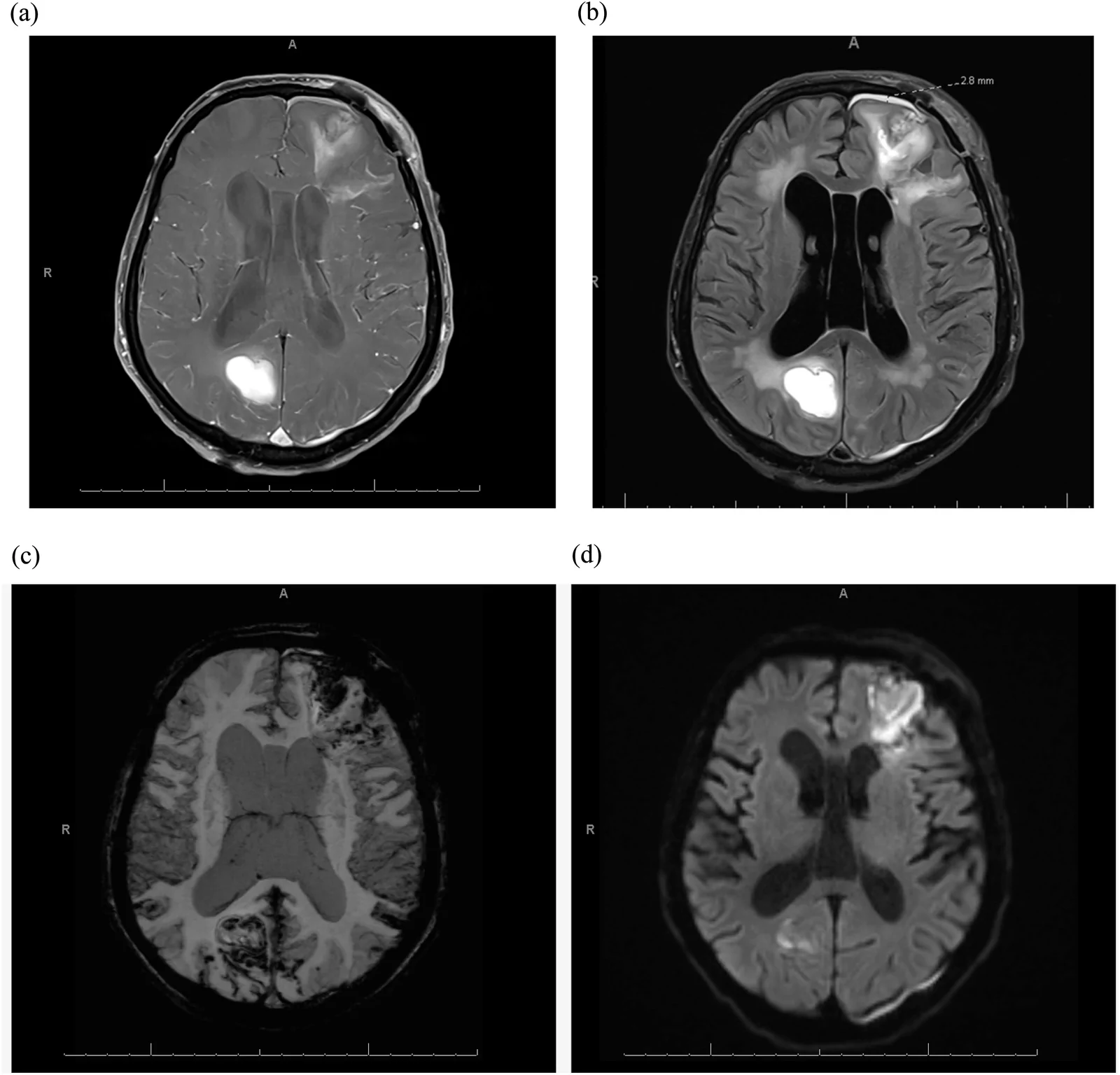
Follow-up MRI and susceptibility findings on 12/02/2025. (A) Follow-up axial post-contrast T1-weighted MRI demonstrated interval evolution of the left frontal intraparenchymal hematoma with cavity-margin enhancement. (B) Axial FLAIR MRI demonstrated increased bilateral convexity subarachnoid blood products compared with the prior MRI. (C) Axial susceptibility-weighted MRI demonstrated multifocal susceptibility effects corresponding to evolving intraparenchymal and extra-axial blood products. (D) Diffusion-weighted MRI demonstrated several new punctate areas of restricted diffusion involving the superior aspects of both cerebral hemispheres, right greater than left

Histopathologic examination of resected tissue showed cerebral and leptomeningeal vessels containing Congo red-positive amyloid with apple-green birefringence under polarized light, accompanied by transmural lymphocytic and macrophage-predominant inflammation, fibrinoid necrosis, and reactive gliosis (Figure 2). No well-formed granulomas were identified, however, the fragmented hematoma evacuation specimen limited assessment of vascular architecture. Immunohistochemistry demonstrated T-cell (CD3+, CD4+) and macrophage (CD68+) infiltration, consistent with amyloid-β–related angiitis. Subsequent brain MRI revealed evolving hematomas with progressive vasogenic edema, new multifocal punctate ischemic infarcts, increasing cortical microbleeds, and convexity subarachnoid hemorrhage, findings suggestive of inflammatory cerebral amyloid angiopathy (Figure 3 and 4). In conjunction with the biopsy results, these findings confirmed the diagnosis of amyloid-β–related angiitis.

Because the presentation was atypical for classic ABRA, an extensive infectious evaluation was performed and was unrevealing. The patient was treated with 5 days of high-dose intravenous methylprednisolone followed by an oral prednisone taper. He demonstrated gradual but clear improvement in mental status and interaction, supporting an immune-mediated process.

After the index hospitalization, the patient required PEG tube placement for post-stroke dysphagia and experienced recurrent infectious complications. He was readmitted 2 months later with sepsis due to pneumonia, later developed a new nonoperable right frontal parenchymal hemorrhage with intraventricular extension and midline shift, was transitioned to comfort-focused care after goals-of-care discussions with family, and ultimately died.

## Discussion

This case illustrates a potential link between thrombolytic therapy and the unmasking or exacerbation of amyloid-β–related angiitis (ABRA), resulting in rapid neurologic deterioration characterized by multifocal hemorrhage, ischemia, and progressive encephalopathy. The combination of evolving lobar hemorrhages, new punctate infarcts, vasogenic edema, cortical microbleeds, and convexity subarachnoid hemorrhage is highly consistent with inflammatory cerebral amyloid angiopathy and has been described in pathologically confirmed cases of ABRA.^3^ In our patient, histopathologic confirmation established ABRA as the unifying mechanism underlying this radiographic and clinical progression.

While thrombolysis remains a cornerstone of acute ischemic stroke treatment, its use in patients with underlying cerebral amyloid angiopathy may carry risks beyond hemorrhagic transformation alone. One proposed mechanism is that thrombolytic therapy disrupts the blood–brain barrier, increasing exposure of vascular amyloid-β to the immune system and triggering an exaggerated inflammatory response. Histopathologic studies in ABRA demonstrate macrophage-mediated phagocytosis of amyloid-β and perivascular T-cell infiltration, supporting an immune-driven process.^4-6^ Similar cases in the literature describe fatal or fulminant hemorrhagic deterioration following thrombolysis in patients later found to have ABRA, suggesting that thrombolysis may precipitate or amplify vascular inflammation in susceptible individuals.^4,5^ Additionally, delayed inflammatory cerebral amyloid angiopathy has been reported after ischemic stroke even without thrombolysis, further supporting the concept that vascular injury or blood–brain barrier disruption can trigger immune activation in amyloid-laden vessels.^6^

From a diagnostic perspective, early imaging findings of ABRA or CAA-related inflammation may be subtle. Patchy white matter edema can be mistaken for chronic small vessel disease or age-related changes, whereas convexity subarachnoid hemorrhage, superficial siderosis, and cortical microbleeds should raise suspicion for underlying cerebral amyloid angiopathy or CAA-related inflammation. Recognition is particularly challenging in the acute stroke setting, when attention is focused on large-vessel occlusion and reperfusion therapy.

Current American Heart Association/American Stroke Association guidelines do not list cerebral amyloid angiopathy as an absolute contraindication to thrombolysis, although they acknowledge increased risk of symptomatic intracerebral hemorrhage in patients with a high cerebral microbleed burden and uncertainty regarding net benefit in this population.^7^ Our case suggests that inflammatory complications such as ABRA may represent an additional, underrecognized mechanism of post-thrombolysis deterioration in patients with underlying amyloid angiopathy.

The decision to administer thrombolysis for suspected CRAO remains an area of clinical uncertainty. CRAO is considered an ophthalmologic and cerebrovascular emergency, and observational data have suggested that early reperfusion may improve visual outcomes in selected patients. However, randomized trial data have not demonstrated a clear benefit of systemic thrombolysis. Studies show that intravenous tenecteplase administered within 4.5 hours of symptom onset does not improve visual outcomes compared with aspirin and was associated with a higher frequency of serious adverse events, including 1 fatal intracranial hemorrhage.^8^ Although these data were published after the clinical event described in this case, they underscore the uncertainty surrounding thrombolysis for isolated CRAO and highlight the importance of individualized risk-benefit discussion, particularly in older patients with imaging markers suggestive of cerebral amyloid angiopathy or prior susceptibility lesions.

Retrospectively, the patient’s prior MRI findings of multifocal susceptibility signal loss raise the question of whether rapid review of prior susceptibility imaging could inform individualized risk-benefit discussions before thrombolysis in selected older patients, particularly when the indication is clinically debatable, or the neurologic deficit is isolated. Routine emergent MRI before thrombolysis is generally not feasible in most acute stroke workflows and should not delay otherwise indicated time-sensitive treatment. However, early MRI after unexpected post-thrombolysis deterioration may help identify evolving features of CAA-related inflammation, including vasogenic edema, new cortical microbleeds, convexity subarachnoid hemorrhage, and punctate infarcts.

## Clinical Implications

Amyloid-β–related angiitis (ABRA) is a rare inflammatory vasculopathy that may present as severe neurologic deterioration after thrombolysis. Unlike the typical subacute presentation of inflammatory cerebral amyloid angiopathy or amyloid-β–related angiitis, which is often preceded by weeks to months of progressive headaches, cognitive decline, behavioral changes, or seizures, our patient had no such prodromal symptoms per family report, and instead developed abrupt neurologic deterioration within 24 hours of thrombolysis. Inflammation superimposed on amyloid angiopathy likely explains the multifocal infarcts, progressive edema, new microbleeds, and recurrent hemorrhages observed in this patient. Although uncommon, similar reports suggest that thrombolysis can unmask or exacerbate CAA-related inflammation in susceptible individuals. Clinicians should maintain a high index of suspicion for ABRA in older patients with unexpected worsening after tPA or TNK, particularly when MRI demonstrates evolving lobar hemorrhage, vasogenic edema, and cortical microbleeds. Early recognition is essential, as prompt immunosuppressive therapy may improve outcomes in this otherwise devastating but potentially treatable condition.
